# Clinical features and seasonality of parechovirus infection in an Asian subtropical city, Hong Kong

**DOI:** 10.1371/journal.pone.0184533

**Published:** 2017-09-08

**Authors:** Grace P. K. Chiang, Zigui Chen, Martin C. W. Chan, Simon H. M. Lee, Angela K. Kwok, Apple C. M. Yeung, E. Anthony S. Nelson, Kam Lun Hon, Ting Fan Leung, Paul K. S. Chan

**Affiliations:** 1 Departments of Paediatrics, Faculty of Medicine, The Chinese University of Hong Kong, Prince of Wales Hospital, Shatin, New Territories, Hong Kong SAR, China; 2 Departments of Microbiology, Faculty of Medicine, The Chinese University of Hong Kong, Prince of Wales Hospital, Shatin, New Territories, Hong Kong SAR, China; University of Hong Kong, HONG KONG

## Abstract

**Background:**

The epidemiology of human parechovirus (HPeV) in Asia remains obscure. We elucidated the prevalence, seasonality, type distribution and clinical presentation of HPeV among children in Hong Kong.

**Methods:**

A 24-month prospective study to detect HPeV in children ≤36 months hospitalized for acute viral illnesses.

**Results:**

2.3% of the 3911 children examined had HPeV infection, with most (87.5%) concentrated in September-January (autumn-winter). 81.3% were HPeV1 and 12.5% were HPeV4, while HPeV3 was rare (2.5%). HPeV was a probable cause of the disease in 47.7% (42/88), mostly self-limiting including acute gastroenteritis, upper respiratory tract infection and maculopapular rash. A neonate developed severe sepsis-like illness with HPeV3 as the only pathogen detected. A high proportion (60.0%) of children coinfected with HPeV and other respiratory virus(es) had acute bronchiolitis or pneumonia. Six children with HPeV coinfections developed convulsion / pallid attack. Most rash illnesses exhibited a generalized maculopapular pattern involving the trunk and limbs, and were more likely associated with HPeV4 compared to other syndrome groups (36.4% vs. 3.1%, p = 0.011).

**Conclusions:**

In Hong Kong, HPeV exhibits a clear seasonality (autumn-winter) and was found in a small proportion (2.3%) of young children (≤36 months) admitted with features of acute viral illnesses. The clinical presentation ranged from mild gastroenteritis, upper respiratory tract infection and febrile rash to convulsion and severe sepsis-like illness. HPeV3, which is reported to associate with more severe disease in neonates, is rare in Hong Kong. HPeV coinfection might associate with convulsion and aggravate other respiratory tract infections.

## Introduction

Human parechoviruses (HPeV), a group of non-enveloped, positive-sense, single-stranded RNA viruses, were first classified as a distinct genus in 1990s [1]. HPeV are fastidious to grow and escape detection by PCR that is based on pan-enterovirus primers. Most routine laboratories do not offer testing for HPeV, and therefore many aspects of epidemiological and clinical features of this ubiquitous virus remain unknown.

Based on phylogenetic analysis of the VP1 encoding region, 16 genotypes of HPeV have been identified with HPeV1-8 accounting for the majority of infections described in the literature. Current data indicate that HPeV1 is the most common type, and it appears to associate with a certain portion of mild gastrointestinal illnesses and respiratory tract infections. HPeV3 is more often linked with severe diseases including sepsis-like illness and meningoencephalitis, especially in neonates and young infants of less than 3 months old [2–6]. HPeV3 has also been linked with apnoea in premature infants. HPeV encephalitis often occurs in young infants and presents with seizure and diffuse restriction on MRI. While the short-term outcome is reassuring, it is associated with neurodevelopment sequelae. Overall, sepsis-like illness is often associated with HPeV3, but cases linked with HPeV4 have been reported. The spectrum of clinical presentation of HPeV infection appears to be wide including myocarditis, haemolytic uraemic syndrome, necrotizing enterocolitis, acute flaccid paralysis, myalgia and haemophagocytic lymphohistiocytosis [1–7]. Due to the fact that asymptomatic shedding of HPeV is very common in young children, an aetiological role of HPeV in some of these conditions has yet to be firmly established.

## Methods

### Study location

The study was conducted in Hong Kong, a small densely populated cosmopolitan city of 7.2 million people located on the coast of Southern China (22.3^o^N, 114.2^o^E). Hong Kong has a subtropical climate. January-February are the coldest months, with temperatures occasionally below 10°C in the urban areas. March-April are the spring months with relatively higher humidity. May–September are hot and humid with afternoon temperatures often exceeding 31°C. October–December are dry with comfortable temperatures around 15–25°C.

### Patient recruitment

A 24-month study was conducted at a general district hospital with a catchment population of about 650,000, including about 25,000 children under 5 years old. Children aged ≤36 months admitted for suspected acute viral illnesses were identified. To achieve an even recruitment of subjects over the study period, an age- and time-stratified random selection approach was adopted.

In this teaching hospital, children with features of respiratory tract infection were routinely tested for influenza A and B, respiratory syncytial virus (RSV), adenovirus, parainfluenza virus types 1, 2 and 3, enterovirus, rhinovirus and metapneumovirus, while those with acute gastroenteritis were tested for norovirus and rotavirus. Furthermore, comprehensive bacterial investigations were performed when clinically indicated. All these routine tests were conducted according to accredited methods. For the purpose of this study, the specimens were also tested for HPeV regardless of the results of other investigations. The study was approved by the Joint Chinese University of Hong Kong–New Territories East Cluster Clinical Research Ethics Committee. Consent from individual parent for participating in this study was waived by the ethics committee as the study only involved testing the reminder of samples that had been collected for diagnostic purpose under clinical indication. No additional sample or procedure would be collected or performed, and all personal identifiable data were removed from study analysis. The decision on collection of CSF for investigation was clinical and it was not altered by this observational study. Basically, it was a holistic decision considering the clinical presentation, suspicion on pathology of the central nervous system, findings from investigation of other specimens, and the potential clinical value of CSF investigation.

### HPeV detection and typing

Viral RNA was extracted with PureLink® Viral RNA/DNA Mini Kit (Life Technologies). HPeV RNA was detected by real-time PCR using primers (PeV-F: 5’-WGC CYC TGG GSC CAA AAG-3’, PeV-R: 5’-GGC CCC WGR TCA GAT CCA YAG-3’) and probe (FAM-ATG CCC WGR AGG TAC C-MGB) targeting the conserved region of the 5’untranslated region (5’UTR), adopted from a previous study [8]. Real-Time PCR was performed using SuperScript® III Platinum® One-Step Quantitative RT-PCR System with ROX (Life Technologies). A 25-μL reaction mixture contained 12.5 μL of 2X reaction mix, 0.25 μL of SuperScript® III RT/Platinum® *Taq* Mix, primers, probe and 2 μL of specimen RNA sample. The primer and probe concentrations were 400 nM and 200 nM, respectively. The RT-PCR thermal profile consisted of an initial RT step of 15 min at 50°C, followed by 2 min at 95°C, then 40 cycles of 15 sec at 95°C and 1 min at 58°C. Amplification, detection and data analysis were performed with StepOnePlus™ Real-Time PCR System (Life Technologies). Samples positive for HPeV RNA were subjected to sequencing of the capsid protein VP1 [9].

HPeV genotype was assigned based on the maximum likelihood (ML) tree topology constructed using RAxML MPI v8.2.8 [10], based on the global nucleotide alignment of the VP1 gene sequences.

### Diarrhoea virus detection

Children with symptoms of acute gastroenteritis were tested for rotavirus and norovirus on a routine basis. For the purpose of this study, stool samples positive for HPeV were subjected to multiplex PCR as described previously [11], which is capable of detecting viruses associated with acute gastroenteritis including rotavirus, norovirus, adenovirus 40/41, sapovirus, astrovirus, and aichi virus.

### Human herpesvirus 6 detection

Children with rash illnesses were tested for Human Herpesvirus 6 (HHV-6) DNA by real-time PCR using a previously described method [12].

### Statistical analyses

Distributions in age were compared using Mann-Whitney U test. Proportions of HPeV types were compared by Fisher’s Exact test. Statistics were performed with SPSS (IBM® Version 20) or StatCalc (Epi Info^®^, CDC). Two-tailed p-values of ≤0.05 were regarded as statistically significant.

## Results

### Study subjects and specimens

3911 children were recruited during the study period (March 2014-Feb 2016), with 129–255 children examined per month. The male-to-female ratio was 1.3:1, and 7.3% were neonate, 37.0% were 4 weeks to <12 months, 30.4% were 12-<24 months, and 25.4% were 24-<36 months.

A total of 4567 specimens collected from the 3911 children were tested for HPeV: 57.0% were nasopharyngeal aspirate samples or flocked swabs (NPA/NPFS); 32.5% were stool samples or rectal swabs; 4.4% were cerebrospinal fluid; and 4.9% were blood (Table 1). The positive rate for stool / rectal swab was higher than NPA/NPFS (4.4 vs. 1.4%). Only two blood samples were positive, and both were from children who had parechovirus HPeV from stool / NPA as well.

**Table 1:** Parechovirus detection rate according to specimen type.

No. of specimen tested (N)	Parechovirus positive, N (%)^1^	Type distribution, N (%)^2^						
		HPeV1	HPeV2	HPeV3	HPeV4	HPeV5	HPeV6	NA
**Nasopharyngeal aspirate / flocked swab (2604)**	36 (1.4)	26 (72.2)	0	1 (2.8)	0	0	0	9 (25.0)
**Stool / rectal swab (1485)**	66 (4.4)	50 (75.8)	0	1 (1.5)	10 (15.2)	1 (1.5)	2 (3.0)	2 (3.0)
**Blood (226)**	2 (0.9)^3^	1 (50.0)	0	0	1 (50.0)	0	0	0
**Cerebrospinal fluid (201)**	0	—	—	—	—	—	—	—
**Nasal / throat swab (44)**	0	—	—	—	—	—	—	—
**Eye / oral swab (5)**	0	—	—	—	—	—	—	—
**Vesicle fluid (1)**	0	—	—	—	—	—	—	—
**Urine (1)**	0	—	—	—	—	—	—	—
**Total (4567)**	104 (2.3)	77 (74.0)	0	2 (1.9)	11 (10.6)	1 (1.0)	2 (1.9)	11 (10.6)

NA, typing result not available because of insufficient DNA quality for sequencing

^1^ No. of specimen tested as denominator.

^2^ No. of parechovirus-positive specimen as denominator.

^3^ Includes a 10-month-old girl (case no. 5) presented with gastroenteritis and parechovirus type 1 was also detected in stool and NPA, and the stool sample was also positive for norovirus. The other 20-month-old boy (case no. 57) presented with rash over forehead, erythematous periorbital swelling and fever, and parechovirus type 4 was also detected in stool, and the blood sample was also positive for human herpesvirus 6.

### Demographic

Overall 88 children had HPeV detected in their specimens, with 49 (55.7%) boys and 39 44.3%) girls, 3 (3.4%) neonates, 40 (45.5%) aged 4 weeks to <12 months, 29 (33.0%) aged 12-<24 months, and 16 (18.2%) aged 24-<36 months (S1 Table).

The majority (59.1%, 52/88) of the HPeV-infected children had viruses detected from stool or rectal swabs, 25.0% (22/88) from NPA and 15.9% (14/88) from both stool and NPA (S1 Table).

### Seasonality

**Fig 1:**
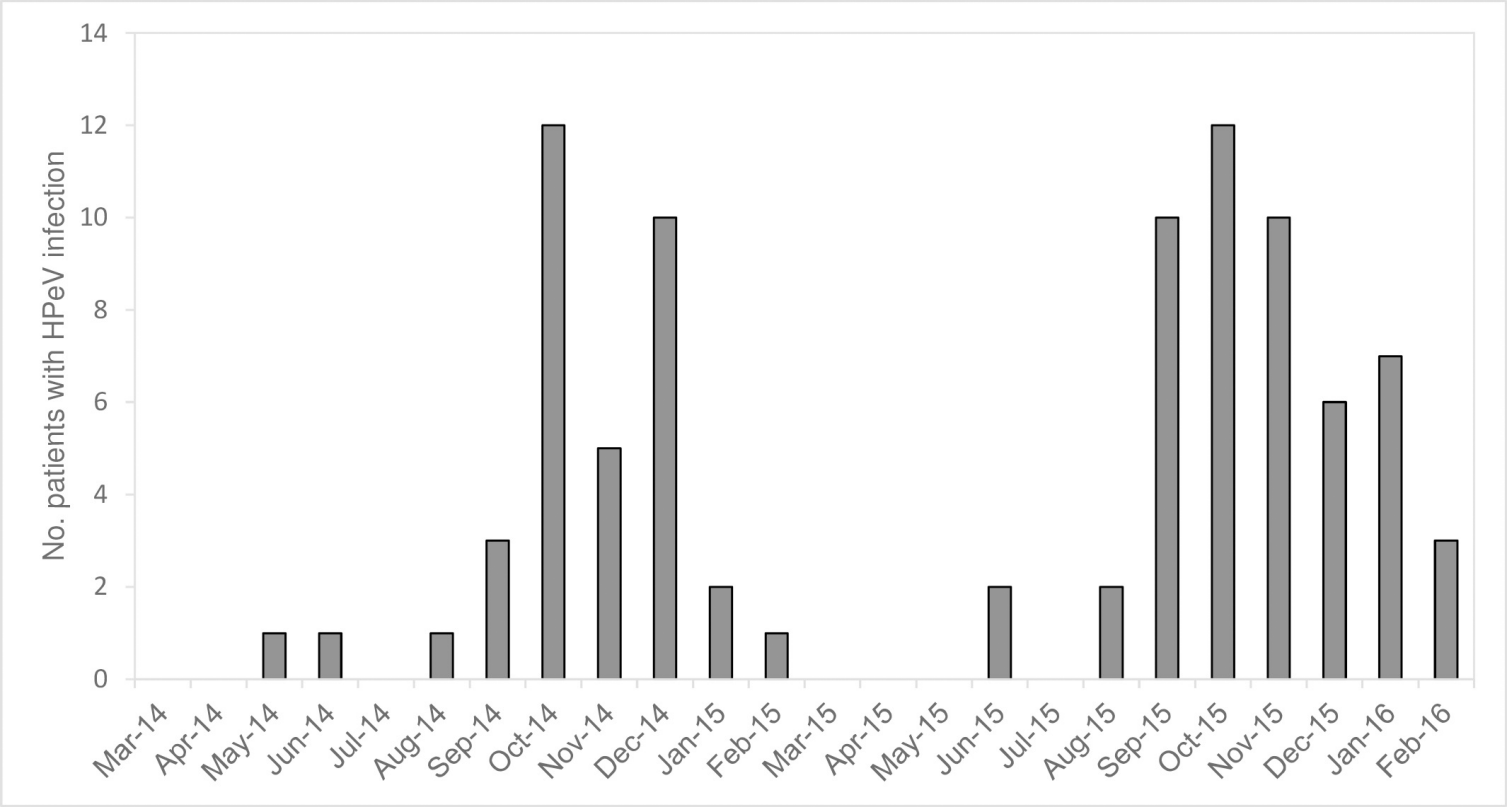
Temporal distribution of human parechovirus (HPeV) infections in Hong Kong.

A sharp seasonality was observed with the majority (77/88, 87.5%) of infections detected between September and January, corresponding to the autumn and winter seasons in Hong Kong (Fig 1). The same seasonal pattern was observed in both years.

### Type distribution

**Fig 2:**
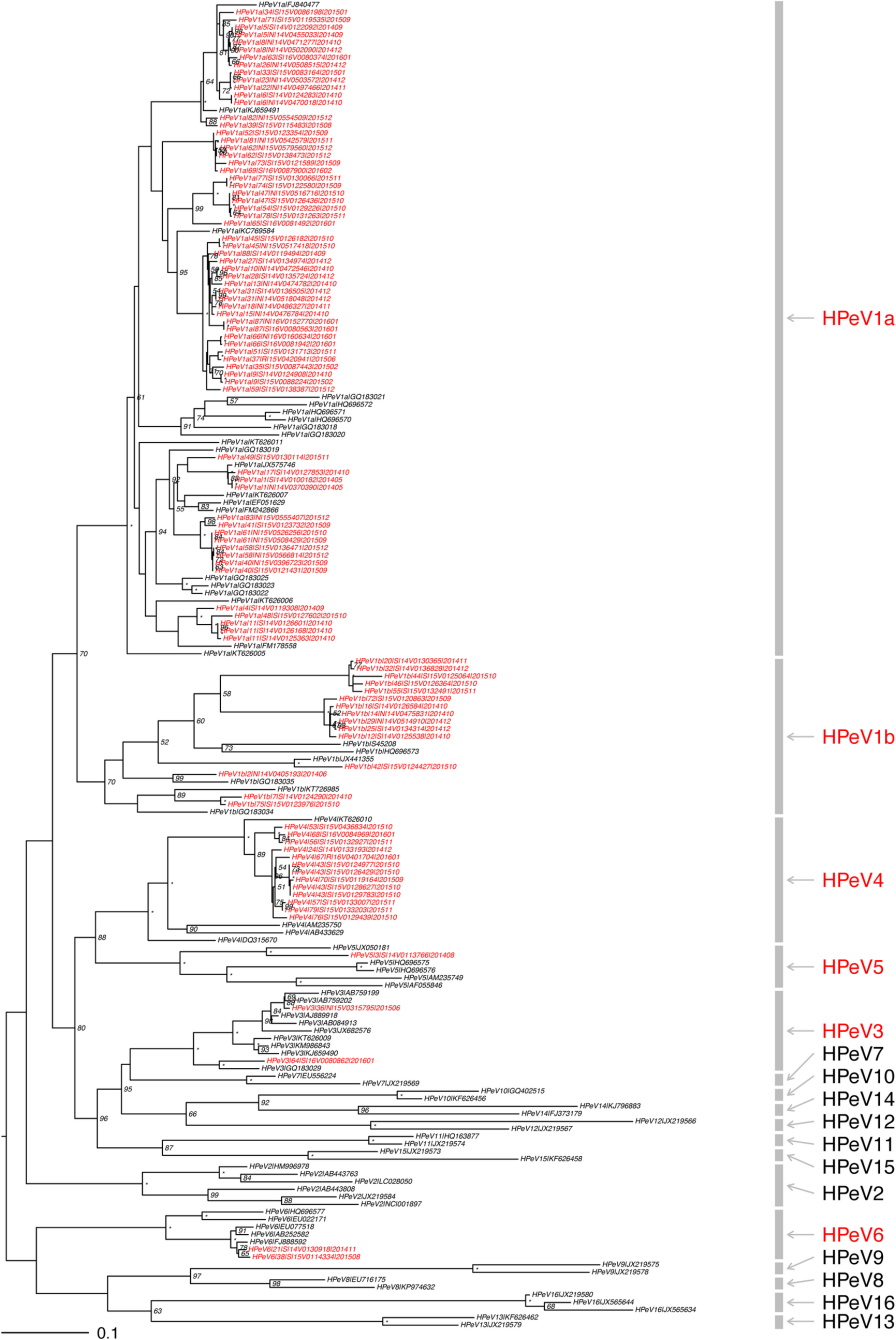
Maximum likelihood (ML) phylogenetic tree of human parechoviruses (HPeV) detected in Hong Kong. Maximum likelihood tree constructed using RAxML MPI v8.2.8, based on the global nucleotide sequence alignment of the VP1 gene of HPeV isolates collected in this study, the represented complete genomes of HPeV1-8, and the VP1 gene of HPeV9-16. Numbers beside of branch nodes indicate bootstrap supports by RAxML; an asterisk (*) indicates 100% support while the index less than 50% was not shown in the tree. The Hong Kong isolates indicated in red were assigned to specific type based on the tree topology. The bar scale at the bottom of the tree indicates percent variation per unit length. The VP1 gene nucleotide sequences obtained in this study were submitted to NCBI/GenBank database, with accession numbers of MF435179—MF435277. “16V0080862” and “15V0315795” were the HPeV3 strains detected in this study. “15V0315795” close to the Yamagata 2011 lineage (AB759199) was from a 6-day neonate with sepsis-like illness, while the “16V0080862” close to the Netherlands 2006 lineage (GQ183029) was from a 7-month girl with acute gastroenteritis. Both children were not coinfected with other pathogens.

Of the 88 HPeV-infected children, 90.9% had samples with sufficient quality for virus type identification. The phylogenetic tree of VP1 sequences is shown in Fig 2. HPeV1 predominated and accounted for 81.3% (65/80) of infections, followed by HPeV4 (12.5%, 10/80). Other HPeV types were rare: HPeV3 (2 cases), HPeV6 (2 cases), and HPeV5 (1 case).

#### Age

Children with HPeV1 infection appeared older than those infected with non-type 1 HPeV (median age [interquartile range]: 12 [9–20] months vs. 8 [7–19.5] months). However, it did not reach statistical significance (p = 0.184 by Mann-Whitney U test) (Table 2).

**Table 2:** Age distribution of children according to the type of human parechovirus (HPeV).

Age groups	No. of children positive for HPeV (%)*			
	All types combined	HPeV1	HPeV4	HPeV3/5/6
<4 weeks	2 (2.5)	1 (1.5)	0 (0)	1 (20.0)
4 weeks–<12 months	38 (47.5)	30 (46.2)	6 (60.0)	2 (40.0)
12 –<24 months	27 (33.8)	22 (33.8)	3 (3.0)	2 (40.0)
24 –<36 months	13 (16.3)	12 (18.5)	1 (10.0)	0 (0)
Total	80	65	10	5

*Not include 8 children who were positive for HPeV by real-time PCR but with insufficient viral RNA quality for type identification.

#### Specimen

The spectrum of HPeV types found in NPA was less diverse than those found in stool / rectal swab samples. Of the 27 NPA with virus type identified, only 1 (3.7%) was non-type 1 HPeV, whereas 14 (21.9%) of the 64 typable stool / rectal swab specimens were non-type 1 HPeV (p = 0.03 by Fisher’s Exact test) (Table 1).

### Clinical presentation

**Fig 3:**
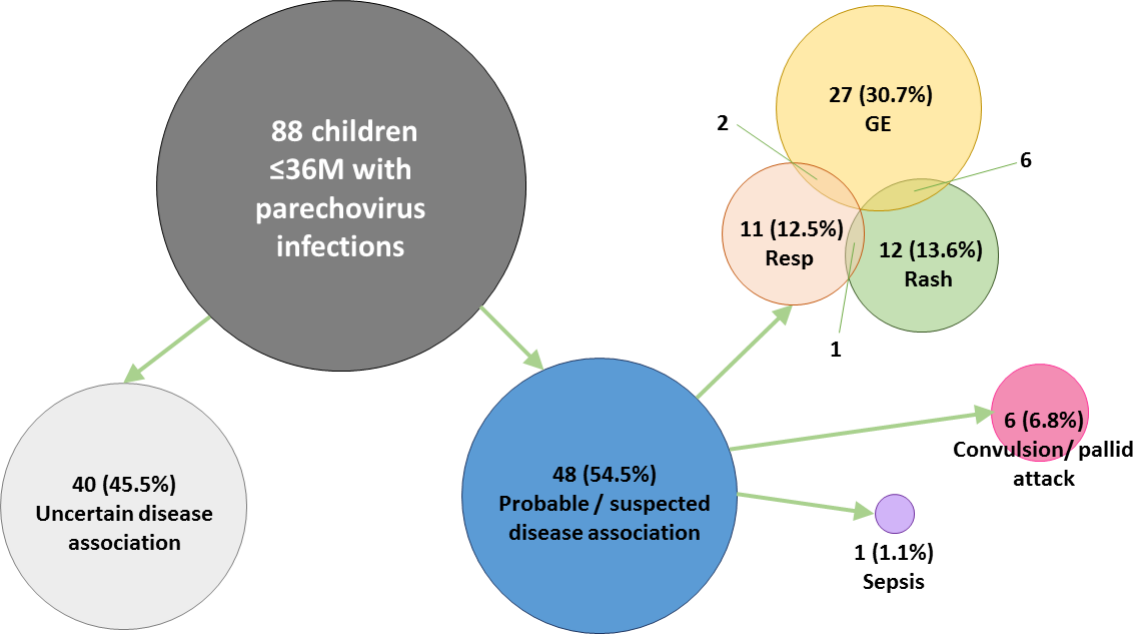
Overview on major illnesses of 88 children with human parechovirus (HPeV) infection. GE: acute gastroenteritis. Resp: acute respiratory illness. All 88 children included had HPeV RNA detected from one or more specimens. Cases without coinfecting pathogen that could have accounted for the illness were considered as having a “probable” disease association with HPeV. Six children with convulsion / pallid attack and were coinfected with pathogen not typically associated with neurological manifestation were considered as having a “suspected” disease association with HPeV. “Uncertain” disease association referred to children coinfected with pathogen(s) that typically associate with the presenting illness.

The key findings of 88 children with parechovirus detected are shown in S1 Table. HPeV-infected children were grouped according to the major system(s) involved and separated into those with HPeV being the only infection detected versus those with other pathogen(s) identified. When HPeV was the only infection identified, it was regarded as having a “probable” association with the illness. Whereas when HPeV was detected in association with other conventional pathogens that could well explain the illness, the role of HPeV was regarded as “uncertain”. Since the association between respiratory and gastrointestinal pathogens with neurological presentation is less clear, the role of HPeV was regarded as “suspected” in these cases (Fig 3).

#### HPeV as a “probable” cause of acute gastroenteritis

30.7% (27/88) of HPeV-infected children were regarded as having HPeV being a “probable” cause of the acute gastroenteritis illness based on the fact that no gastroenteritis-associated pathogens, other than HPeV, was identified from stool. Among them, the majority (25/27, 92.6%) had diarrhoeal symptoms and the frequency of diarrhoea varied from once a day to more than 10 times a day. In contrast, vomiting occurred much less frequently (12/27, 44.4%) and was usually mild. Four of these 27 children also had HPeV detected from NPA with two of them also coinfected with parainfluenza type 2 and RSV, respectively, and all had respiratory symptoms. Another seven children had respiratory viruses detected from NPA, and all except one had respiratory symptoms recorded. Six of these 27 children developed rash whose clinical features are described below.

#### HPeV as an “uncertain” cause of acute gastroenteritis

37.5% (30/88) of infected children had symptoms of acute gastroenteritis and with HPeV detected from stool, but the role of HPeV was regarded as “uncertain” based on the fact that other gastroenteritis-associated pathogens (most commonly norovirus, rotavirus, *Salmonella* and *Campylobacter spp*.) were also found in their stool samples. Four of the 30 children from this group also developed rash whose clinical features are described below.

#### HPeV as a “probable” cause of acute respiratory illness

12.5% (11/88) of infected children were regarded as having HPeV being a “probable” cause of the acute respiratory illness based on the fact that no respiratory pathogen, other than HPeV, was identified from NPA. The most common symptoms were cough and runny nose. Five children had mild shortness of breath or noisy breathing, and one developed pneumonia with chest X-ray showing mild left perihilar haziness but without significant consolidation. A substantial proportion (6/11, 54.5%) of them also had HPeV detected from stool. Of these 11 children with probable HPeV-associated acute respiratory illness, five also developed symptoms of acute gastroenteritis with two had coinfection with *Salmonella enteritidis* and *Campylobacter jejuni*, respectively. In another case, the disease association could not be verified as no stool sample was collected. One child developed a rash whose clinical features are described below.

#### HPeV as an “uncertain” cause of acute respiratory illness

Altogether, 20 children with HPeV detected from NPA also had coinfection with other respiratory pathogens including enterovirus/rhinovirus (10), RSV (3), influenza A (1), parainfluenza type 1, 2, and 3 (1 each), adenovirus (1), adenovirus and enterovirus/rhinovirus coinfection (1), adenovirus and parainfluenza type 4 coinfection (1). A large proportion (12/20, 60.0%) of these children with HPeV coinfections developed lower respiratory tract involvement with acute bronchiolitis or pneumonia.

#### HPeV as a “probable” cause of rash illness

Of the 88 HPeV-infected children, 12 (13.6%) were regarded as having HPeV being a “probable” cause of the rash illness based on the fact that no other rash-associated pathogen was identified. In all except one child, HPeV was found from stool/rectal swab (Table 3). The rash presentation was mainly maculopapular and often generalized involving trunk and limbs, and accompanied with fever or developed just after fever had subsided (Table 3). The majority of these children presented with other concurrent illnesses, most commonly acute gastroenteritis and mild upper respiratory tract illness. Six children had other pathogens identified which could account for the concurrent illnesses, but these pathogens were not typically associated with rash.

**Table 3:** Characteristics of children with rash illness.

Case No.	Sex / Age (months)	Presentation of rash	Concurrent illness(es)	Sample(s) positive for HPeV		HPeV type	Coinfections	HHV-6
16	M / 12	Itchy blanchable maculopapular rash over back, spread to trunk, forearms and feet.	GE		Stool	HPeV1	EV/Rhino, non-typhiodal *Salmonella spp*.	Not detected from NPA*
24	M / 7	Mild fine blanchable maculopapular rash over face, trunk, and right scrotum, sparing limbs. Developed after fever subsided. Clinically considered as roseola.	GE, URTI		Stool	HPeV4	-	Not detected from NPA*
27	F / 9	Maculopapular rash over face and body. Developed after fever subsided. Clinically considered as roseola.	GE, URTI		Stool	HPeV1	-	Not detected from NPA*
34	F / 19	Maculopapular rash over buttock and hands, with fever for 3 days.	GE, URTI		Stool	HPeV1	Parainfluenza virus-1	Detected from NPA*
43	M / 6	A few spots of rash over back, face and neck, with fever for 9 days.	GE, URTI		Stool	HPeV4	EV/Rhino, non-typhoidal *Salmonella spp*.	Not detected from NPA*
49	M / 10	Generalized whole body maculopapular rash, with fever for 2 days.	GE, URTI		Stool	HPeV1	-	Not detected from NPA*
50	M / 16	Maculopapular rash over trunk after fever subsided. Clinically considered as roseola.	GE		Stool	typing failed	-	Not tested, only stool sample available
51	F / 3	Eczematous rash over chest, with fever.	GE, bronchiolitis		Stool	HPeV1	Respiratory syncytial virus	Not detected from NPA*
57	M / 20	Blanchable macular rash over forehead, erythematous periorbital swelling, with fever.	URTI		Stool, blood	HPeV4	-	Detected from blood
58	M / 11	Generalized maculopapular rash. Developed after fever subsided. Clinically considered as roseola.	URTI		Stool, NPA	HPeV1	-	Detected from NPA*
67	F / 20	Itchy rash over trunk and spread to limbs and scalp, weeping, crusting, peeling and sandpaper-like. Features of cellulitis. No fever.	-		Rectal swab	HPeV4	*Staphylococcus aureus* from skin swab	Not detected from blood
68	M / 8	Eczematous rash, with fever.	GE, croup, pneumonia		Stool	HPeV4	-	Not detected from NPA*
76	F / 7	A few patches of maculopapular rash over right hand only, no fever.	GE, URTI		Stool	HPeV4	Norovirus, EV/Rhino	Not detected from NPA*
77	F / 7	On and off skin rash, faint scattered spots over face and trunk.	GE		Stool, NPA	HPeV1	Norovirus	Not detected from blood
81	M / 16	Mild generalized erythematous patches, with fever.	GE, bronchiolitis		NPA	HPeV1	-	Not detected from NPA*
87	F / 10	Maculopapular rash over trunk. Developed after fever subsided. Clinically considered as roseola.	URTI		Stool, NPA	HPeV1	Parainfluenza virus-1	Not detected from NPA*

HPeV: human parechovirus, NPA: nasopharyngeal aspirate, GE: acute gastroenteritis, URTI: upper respiratory tract illness, HHV-6: human herpesvirus type 6 detection by PCR, EV/Rhino: the PCR assay performed on respiratory samples does not differentiate between enterovirus and rhinovirus.

*Serum sample not available.

#### HPeV as an “uncertain” cause of rash illness

A 20-month old girl developed an itchy, weeping, crusting, sand paper-like rash on the scalp and limbs, without fever. The skin swab was positive for *Staphylococcus aureus* which was compatible with clinical presentation. The HPeV4 found in the stool of this child was likely to be a bystander.

Another three children had HHV-6 DNA detected from NPA or blood samples, and one of them was clinically considered as roseola. The role of parechovirus in these was regarded as uncertain.

#### HPeV as a “suspected” cause of neurological illness

Six (6.8%) of the 88 HPeV-infected children developed neurological illnesses including five with convulsion and one with pallid attack. All of these children had concurrent illnesses which could be explained by the coinfection identified. Three children had acute gastroenteritis due to norovirus or non-typhoidal *Salmonella spp*. Another three children had upper respiratory tract infections with enterovirus/rhinovirus or parainfluenza type 3 virus. Since association between HPeV as well as those coinfecting pathogens and the neurological presentation in these children could not be verified, HPeV was regarded as a “suspected” pathogen.

#### Sepsis-like illness

Only one of 88 HPeV-infected children presented with sepsis-like illness. She was a 6-day-old baby girl born full term by vaginal delivery. She presented with fever up to 38.6°C. She did not have any respiratory or gastrointestinal symptoms. Her heart rate was 200 min^-1^ upon admission. She was given an intravenous bolus of normal saline of 40 mL/kg body weight but still had a persistent tachycardia and metabolic acidosis despite her blood pressure being maintained in the normal range. She was admitted to the neonatal intensive care unit for monitoring and empirically treated with intravenous ampicillin and amikacin after sepsis work up. Flagyl was added due to abdominal distension with the worry of abdominal sepsis. Despite that the presentation of impending shock, her infection markers including white cell count and C-reactive protein were not raised. Ultrasound examination of her abdomen was unremarkable and blood, urine and cerebrospinal fluid culture were all negative. Chest X-ray only showed mild bilateral lung field streakiness. Her NPA was positive for HPeV3 which was the only pathogen identified.

#### Age, virus type and clinical presentation

**Fig 4:**
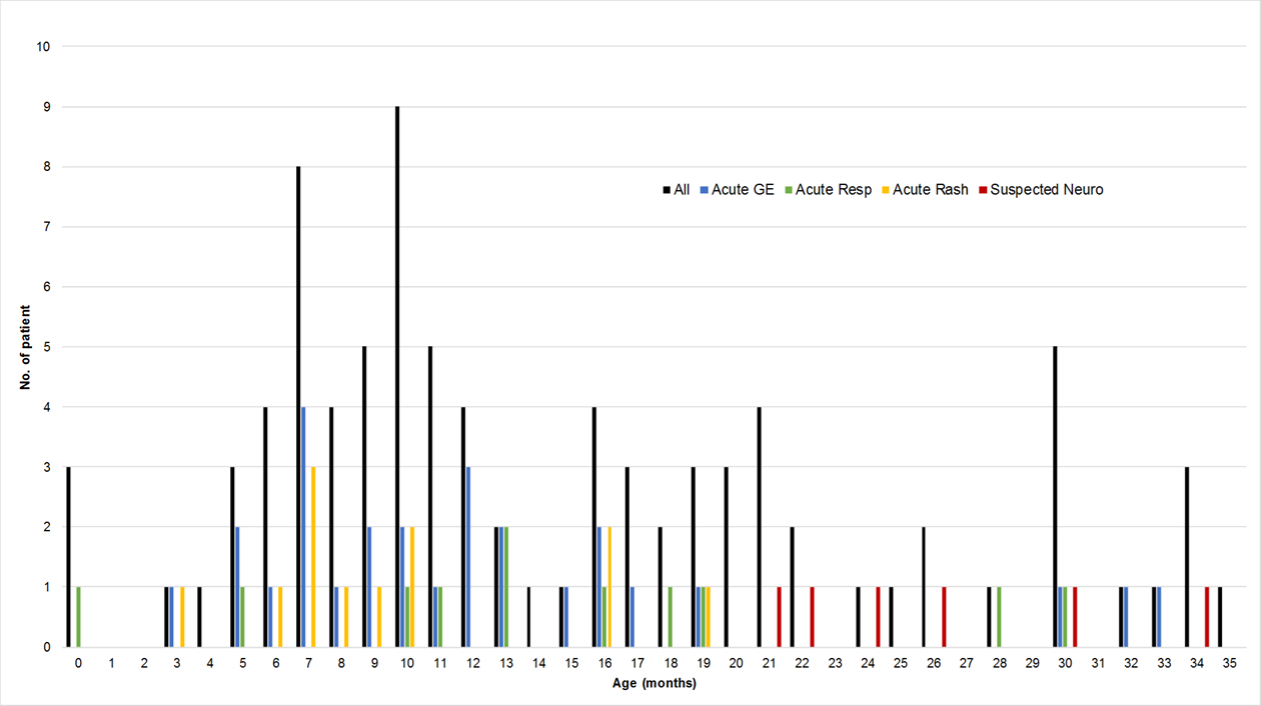
Age distribution of children with human parechovirus (HPeV) infection. All: all 88 children infected with HPeV. Acute GE: 27 children with HPeV regarded as a “probable” cause of acute gastroenteritis. Acute Resp: 11 children with HPeV infection regarded as a “probable” cause of acute respiratory illness. Acute Rash: 12 children with HPeV infection regarded as a “probable” cause of rash illness. Suspected Neuro: 6 children developed convulsion or pallid attack with HPeV as a coinfection with other pathogen(s).

The age distribution according to clinical presentation is shown in Fig 4. The six children presenting with neurological illness where HPeV was regarded as the “suspected” cause were significantly older (median [interquartile range] months: 25 [22.5–29.0]) than other groups who presented with acute gastroenteritis (11.0 [7–15.5]), acute respiratory illness (13.0 [10.5–18.5]), and rash (8.5 [7–10.5]) (all p-value ≤ 0.01 by Mann-Whitely U test).

**Fig 5:**
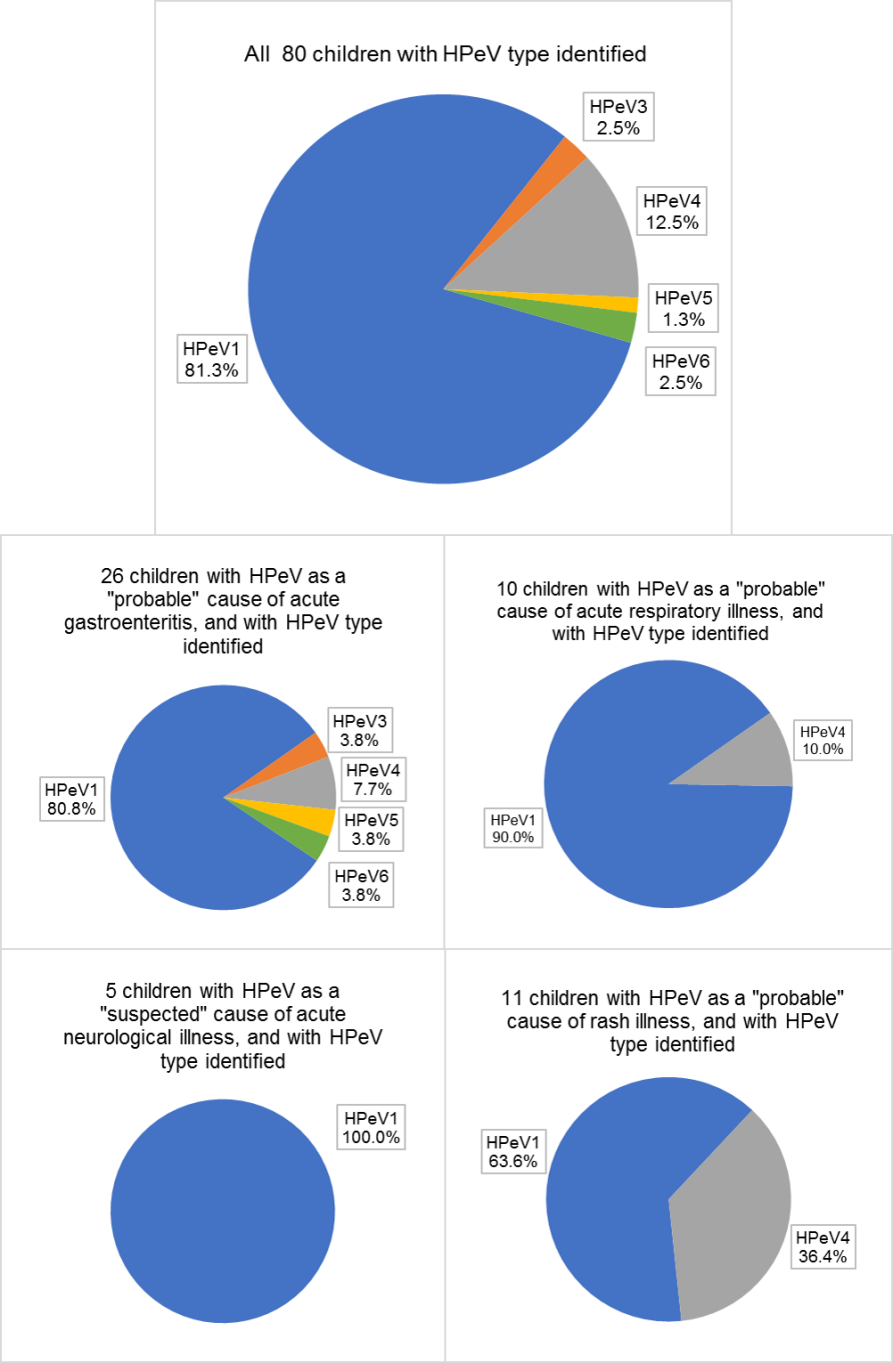
Distribution of human parechovirus (HPeV) types according to clinical presentation.

The distribution of HPeV types according to clinical presentation is shown in Fig 5. Subgroup analysis on the 45 children with HPeV considered as a “probable” cause of illness and with the virus type successfully identified revealed that HPeV4 was significantly more prevalent among children with rash illness compared to those without (36.4% vs. 3.1%, p = 0.011 by Fisher’s Exact test).

## Discussion

The true impact of HPeV infection to human health remains to be defined. With the increased availability of molecular diagnosis in routine service laboratories, the detection of this group of largely non-cultivable viruses has become possible. Serological data indicates that over 90% of children have been infected with HPeV by two years of age [13–15]. Our study showed that about 2.3% of children hospitalized for features of acute viral illnesses in Hong Kong harboured HPeV. While the true aetiological role of HPeV infection is difficult to verify, we estimated that about half (54.5%) of these HPeV infections could have contributed to the presenting illness. Our observed prevalence is in line with the reported range from children with gastroenteritis in Korea (2%) [16] and Hong Kong (2.9%) [17], and children with acute illnesses in Spain (2%) [18] and Geneva (2.6%) [19]. Of note, a few studies had reported very high rate of HPeV detection: 16% from children with enteritis in Salvador, Brazil [20], and 55% from children with diarrhoea in Shanghai, China [21].

While a seasonal pattern of HPeV infection has been observed from most studies, the peak seasons vary considerably, and is in general similar to that of enteroviruses [22]. We found a distinct seasonal pattern with more than 88% of infections detected from September to January corresponding to autumn and winter periods in Hong Kong. However, this is in contrast to the seasonality of enteroviruses in Hong Kong which occurs in summer and autumn.

In this study, all children were thoroughly investigated for possible bacterial and viral pathogens. In particular, a multiplex PCR covering several gastroenteritis-associated viruses was applied to children with HPeV detected from stool. Therefore, this study was able to detected most, if not all, coinfections. As a result, we found that about half of the HPeV-infected children did not have any coinfection, and thus HPeV was considered as the probable cause of the illness. Mild acute gastroenteritis was the most common presentation seen in about two-thirds of these children, followed by upper respiratory tract infection and rash. Severe infection presenting as sepsis-like illness only occurred in one case.

We found that the majority (81.3%) of infections belonged to HPeV1, followed by HPeV4 (12.5%). This pattern is very similar to that reported from a neighbouring city, Guangzhou [23] where travel between the two places is very frequent. On the other hand, HPeV3, the most or second most common type found in Europe and a few Asian cities was rarely (2/80, 2.5%) detected in Hong Kong [24–26]. This may have implications on our local disease burden as it has been shown that HPeV1 is more likely to associate with mild gastroenteritis [27], whereas HPeV3 is more likely to associate with severe diseases including sepsis-like illness, meningoencephalitis and myocarditis in young infants below the age of 3 months [6, 28–32]. In this study we only found two cases of HPeV3 infection. One of them was a 6-day-old neonate presenting with sepsis-like illness, and no pathogen other than HPeV3 was identified. The other case was a 7-month baby girl presenting with acute gastroenteritis, and again no other pathogen was identified. It has been suggested that the higher prevalence of HPeV3 infection among young infants was due to the lack of maternal antibody. It would be worthwhile to further investigate whether the prevalence of HPeV3-specific antibody in adults is higher in Hong Kong and the surrounding region.

A relatively high prevalence of HPeV4 is a remarkable epidemiological feature of Hong Kong, which also seems to be the case for the nearby cities. We found that HPeV4 occurred more frequently in children with rash illness. A characteristic pattern of rash with palmar-plantar distribution has been described in young infants infected with HPeV3 [33–35]. However, the presentation of rash illness associated with other HPeV types, or in older infants and young children is less well defined. In the current study, most of the cases developed a generalized maculopapular rash mainly involving face and limbs, and accompanied with fever. However, the presentation in a few cases was also compatible with roseola, which is associated with HHV-6 infection that is common in young children.

We found six HPeV-infected children developed convulsion and pallid attack. Although all of them had other current illnesses which were explained by the coinfecting pathogens, the role of HPeV remains suspicious. Of note, these children were older than other HPeV infected children who did not have neurological manifestations.

We suspect coinfection of HPeV with other respiratory viruses may confer a higher chance of low respiratory tract involvement, as in the current study, 60% of these children had acute bronchiolitis or pneumonia.

## Limitations

This study was limited by the short duration of 24 months where some rare diseases such as myocarditis and meningoencephalitis [30] might not be revealed. Due to ethical issues, only samples that had been collected under clinical indication could be used for this study. This may have jeopardised the differentiation between truly pathogenic and bystander infections. We therefore cautiously described the association as “probable” or “suspected”.

At this stage, it is difficult to ascertain whether the more diverse distribution in parechovirus types observed in stool samples compared to respiratory specimens was due to type-specific difference in tropism or biases associated with differences in viral load and sensitivity of detection.

While VP1 is a commonly used gene region for identifying the genotype of HPeV, one should be aware of the possibility of recombination leading to misidentification of genotypes.

## Conclusions

HPeV infection had a clear seasonality in Hong Kong, and was found in about 2.3% of young children hospitalized for suspected acute viral illnesses. In about half of them, HPeV had probably contributed to the disease, most commonly acute mild acute gastroenteritis, upper respiratory tract infection and generalized maculopapular rash. Severe sepsis-like illness occurred, though rare. Further study is needed to clarify the role of HPeV coinfection in causing convulsion and aggravating other respiratory viral infections.

## Supporting information

S1 Table List of 88 children with parechovirus detected from clinical specimens. (XLSX)
